# Person-centred care in individuals with stroke: a qualitative study using in-depth interviews

**DOI:** 10.1080/07853890.2022.2105393

**Published:** 2022-08-05

**Authors:** María Belén Martín-Sanz, Rosa María Salazar-de-la-Guerra, Juan Nicolas Cuenca-Zaldivar, María Salcedo-Perez-Juana, Cristina Garcia-Bravo, Domingo Palacios-Ceña

**Affiliations:** a Research Group of Humanities and Qualitative Research in Health Science of Universidad Rey Juan Carlos (Hum&QRinHS), Department of Physical Therapy, Occupational Therapy, Physical Medicine and Rehabilitation, Universidad Rey Juan Carlos, Alcorcón, Spain; b Hospital Management Department, Hospital de Guadarrama, Servicio Madrileño de Salud (SERMAS), Madrid, Spain; c Research Group in Nursing and Health Care, Puerta de Hierro Health Research Institute-Segovia de Arana (IDIPHISA), Madrid, Spain; d Research Group in Evaluation and Assessment of Capacity, Functionality and Disability of Universidad Rey Juan Carlos (TO + IDI), Department of Physical Therapy, Occupational Therapy, Physical Medicine and Rehabilitation, Universidad Rey Juan Carlos, Alcorcón, Spain

**Keywords:** Occupational therapy, physical therapy, patient-centred care, qualitative research, rehabilitation, stroke

## Abstract

**Background:**

Person-centred care (PCC) has considerable effects on the clinical practice of health professionals. The purpose of this study was to describe the perspectives and perceived barriers and enablers of individuals with stroke regarding the PCC model in stroke rehabilitation.

**Methods:**

A qualitative exploratory study was conducted based on an interpretive framework. Participants were recruited using non-probabilistic purposeful sampling and a snowball-technique strategy. The inclusion criteria consisted of: (a) individuals > 18 years, (b) diagnosed with moderate or severe stroke according to the National Institutes of Health Stroke Scale and (c) in the post-acute or chronic stage of the disease. In total, 31 individuals with stroke were included. In-depth interviews and researchers’ field notes were used to collect the data. A thematic analysis was performed. Also, credibility, transferability, dependability and confirmability techniques were followed to establish trustworthiness of the data.

**Results:**

Thirty-one individuals with stroke (11 women) were included. Three main themes were identified: (a) The person behind the “patient” label, recognizing the person beyond their illness and valuing their identity and individual characteristics, (b) The person at the centre of care, considering themselves as an active agent in their own care and respecting their preferences and expectations for their care process and (c) Training for PCC, providing health professionals with tools to achieve professional skills for the implementation and development of the PCC model.

**Conclusions and significance:**

This paper describes relevant aspects that health professionals should consider when providing PCC in the context of the rehabilitation of individuals with stroke.

**Key messages**
- The individuals’ perspective regarding person-centred care (PCC) has considerable effects on the clinical practice of health professionals.
- Individuals with stroke describe how there is a person behind the "patient" label, with identity, needs and desire to participate in decision making.
- Training in the PCC model helps healthcare professionals identify the needs of individuals with stroke during rehabilitation.

## Introduction

Person-centred care (PCC) has gained importance in the context of clinical care over the past few decades [1,2], although certain difficulties still exist in terms of its integration into health systems [3]. Thus, the World Health Organisation considers the humanization of health care and the development of person-centred health systems as key aspects at the core of health care [1], which are fundamental for improving the experience of patients in terms of their care [3]. Therefore, health care policies such as health service planning should focus on the person as a significant element in the evolution of health care [1]. In turn, a systematic review of randomized clinical trials shows that the effects of a person-centred practice carried out by occupational therapists in individuals with stroke help people to accept new limitations and improve satisfaction with occupational performance [4].

The PCC model in healthcare has become synonymous with quality care [5]. There has been a shift from the concept of "patient-centered care" to PCC [6,7]. Although in the patient care model the goal of care is to achieve a functional life, whereas in the PCC model, the goal is to achieve a meaningful life. Therefore, to apply PCC, it is necessary to broaden the perspective and consider the individual's entire life in the clinical context [6], addressing their needs and preferences beyond clinical or medical aspects [7,8] and accepting their complexity [9]. This means that health services should recognize that people with illnesses can work with professionals and participate in decision making, thus fostering empowerment [10].

Despite the growing interest in PCC, there is still no consensus for its definition [3,11] and which factors are decisive for the application of this model in clinical practice [11]. Thus, PPC is characterized by four dimensions [12]; the first is based on the biopsychosocial perspective, considering the person as a whole, focussing on the intimacy, spirituality or beliefs of the sick person; the second describes the sharing of responsibility and power, which includes the decision-making capacity of the person cared for and autonomy, finding common ground in clinical care; the third, treating the individual as a person, including both the disease and the experience of the disease; finally, the fourth describes the strengthening of the therapeutic alliance through the person–physician relationship.

The PCC model includes biomedical variables in care, and the individual's wishes and values in relation to their clinical situation to achieve a better understanding of the effect of treatment and care on the person, their family and environment [7]. In its application, the role of the healthcare professional is essential, because it requires a change in their professional role and their relationship with individuals [13] that can directly influence the development of PCC [14].

Concerning the person with stroke, their health care is seen as a process that requires assessment, feedback and management over time to adapt to changing patient needs [15], so that it is necessary to respect their expectations and values and to work with them in a shared manner [16]. Previous qualitative studies describe the preference of individuals with stroke for PCC [3,17] which includes consideration of their personal wishes, concerns, understanding of their previous circumstances, respect for their routines, future plans and life goals according to their physical, mental and emotional state [15,18–20]. Therefore, PCC is considered a generally accepted goal in post-stroke health care [15]. However, it is necessary to continue defining and describing the components of the PCC model from the perspective of the individuals themselves, within the different clinical contexts [1], assessing the characteristics of the individuals, medical specialties, and the performance of the professionals in the overall care of the person under the PCC approach [21], to generate a real and lasting change in the current approach to care [15]. Qualitative research is useful for describing complex phenomena and understanding the beliefs, values, and motivations that underlie individual health behaviours [22,23]. Furthermore, qualitative studies have been used to research the stroke survivors' experiences and expectations before and after treatment [24] and the individual’s participation in their recovery process [25]. Therefore, what is the meaning of PCC for individuals with stroke? What is their perspective during their rehabilitation? What elements do they consider relevant or critical in the model for their recovery and rehabilitation? The objectives of this study were to describe the perspectives and perceived barriers and enablers of individuals with stroke regarding the PPC model application in stroke rehabilitation.

## Materials and methods

### Study design

A qualitative exploratory study was conducted based on an interpretive framework [22], following the Standards for Reporting Qualitative Research (SRQR) [26] and the Consolidated Criteria for Reporting Qualitative Research (COREQ) [27].

### Ethics

This study was approved by the ethical committee of Universidad Rey Juan Carlos (code: 2106201911119) and the Ethical Committee of Hospital Fundación Hospital Alcorcón (code: 19/69). Participants provided oral informed consent before their inclusion in the study.

### Research team

Before the study, the researchers’ positioning was established *via* two briefing sessions addressing the theoretical framework for this qualitative study, their beliefs and their motivation for the research [26,27]. Six researchers (four women) participated in this study, including one occupational therapist (CGB), three physiotherapists (BMS, JNCZ, MSPJ) and two nurses (RMSG, DPC). All researchers had experience in research in health sciences. None of the researchers had a clinical relationship with the participants, and they did not know each other previously.

### Participants, context and sampling strategies

In this study, a non-probabilistic, purposeful sampling and snowball-technique strategy were used based on relevance to the research question rather than representativeness [22,28]. A purposeful sampling strategy involved deliberately selecting participants [22]. Also, a snowball sampling procedure was applied, in which participants put the researcher in touch with other participants in similar circumstances and who met the inclusion criteria.

The inclusion criteria consisted of: (a) individuals > 18 years, (b) diagnosed with moderate or severe stroke, according to the National Institutes of Health Stroke Scale (NIHSS), [29] (c) individuals who were beyond the acute phase, in sub-acute or chronic stages. The sub-acute stage is defined as the period between the seventh day of the stroke episode and six months [30,31]. The subacute phase is divided into “early” subacute, from day 7 to three months; and “later” sub-acute between three and six months. Finally, the chronic stage is considered after six months [30,31]. The exclusion criteria consisted of: (a) individuals with cognitive decline, and/or with alterations in verbal communication (e.g. aphasia), (b) individuals with mild stroke according to the NIHSS Scale and (c) in the acute stage.

Participants were recruited from two stroke rehabilitation centres. These centres were in the community, outside the hospital setting and received patients from the hospital and other centres who needed to continue rehabilitation. In these centres, there were individuals with stroke who required high-intensity rehabilitation (4–5 h/day of physiotherapy, occupational therapy, speech therapy, and psychology), individuals who required low-intensity treatment (2–3 h/day of physiotherapy and occupational therapy) and those who required sustained rehabilitation aimed at managing sequelae and disability (2–3 days/week of physiotherapy and/or occupational therapy) [32,33]. During recruitment, the study was explained, and information was provided about the research question, PCC and the study design. In qualitative research, a wide variety of proposals exist for justifying and determining sample size [34–36], also, there is no formula for the prior calculation of the sample size, since the results are not intended to be representative and generalizable [22,28]. In this study, the sample size was determined following the proposal by Turner-Bowker *et al.* [37]. These authors reported that 99.3% of concepts, themes and contents emerged with around 30–35 interviews [37]. With this proposal, a greater capacity to identify codes, categories and topics is achieved. In addition, the current proposal also helps researchers to know when to stop collecting data and/or recruiting participants.

Thirty-three individuals with stroke who met the inclusion criteria were contacted and agreed to participate in the study. Of these, two withdrew from the study due to health problems, one of them stated that he did not feel psychologically prepared to be interviewed.

### Data collection

In-depth interviews and researcher’s field notes were used for data collection [22,28]. During the first stage of data collection (participants 1 to 5), participants received unstructured interviews using open questions [28] such as: “Please, can you share your perspective with me regarding PCC during your rehabilitation and recovery process?” Thereafter, the researchers noted the key words and topics identified in the individuals’ responses and used their answers to ask for them to clarify the content [22,28]. After the first five interviews, new areas of inquiry emerged that required further exploration. This led the researchers, by consensus, to decide to include a new phase of data collection through a semi-structured question guide to delve into these new areas of research. The second stage (participants 6–31) consisted of semistructured interviews that were based on a question guide designed to gather information regarding specific topics of interest (Table 1) [22,28]. This question guide was constructed based on accounts obtained from the initial five participants, not based on the perspective or interests of the study investigators. Open-ended follow-up questions were also used to obtain the detailed descriptions. In addition, “Please tell me more about that”, was also used during all the interviews (if needed) to enhance the depth of the discussion surrounding a specific topic.

**Table 1.** Semi-structured interview guide.

Research areas	Questions
To be treated as a person; meaning	During rehabilitation, what does it mean to you to be treated as a person?
The person's background	What aspects of your life as an individual do you consider key in your relationship with healthcare professionals during your rehabilitation?
Elements of PCC^a^	What factors or elements do you think are relevant to the application of PCC by health professionals during rehabilitation?
Barriers and enablers in the application of PCC^a^ during rehabilitation	What barriers can influence the application of PCC^a^ during your rehabilitation? And enablers?
Training to implement PCC^a^	How should health professionals apply PCC^a^ during their rehabilitation? When? What skills, abilities or knowledge should health professionals have in order to apply PCC^a^?

^a^Person-centred care.

The interviews were audio-recorded and transcribed verbatim. Overall, 1273 min of data collection were recorded, with a mean of 41 min (SD 15.8). The interviews were conducted by BMS, MSPJ and CGB and held at a private room at two stroke rehabilitation centres.

Researcher field notes were used as a secondary source of information to provide more in-depth information [28]

### Data analysis

**Figure 1.**
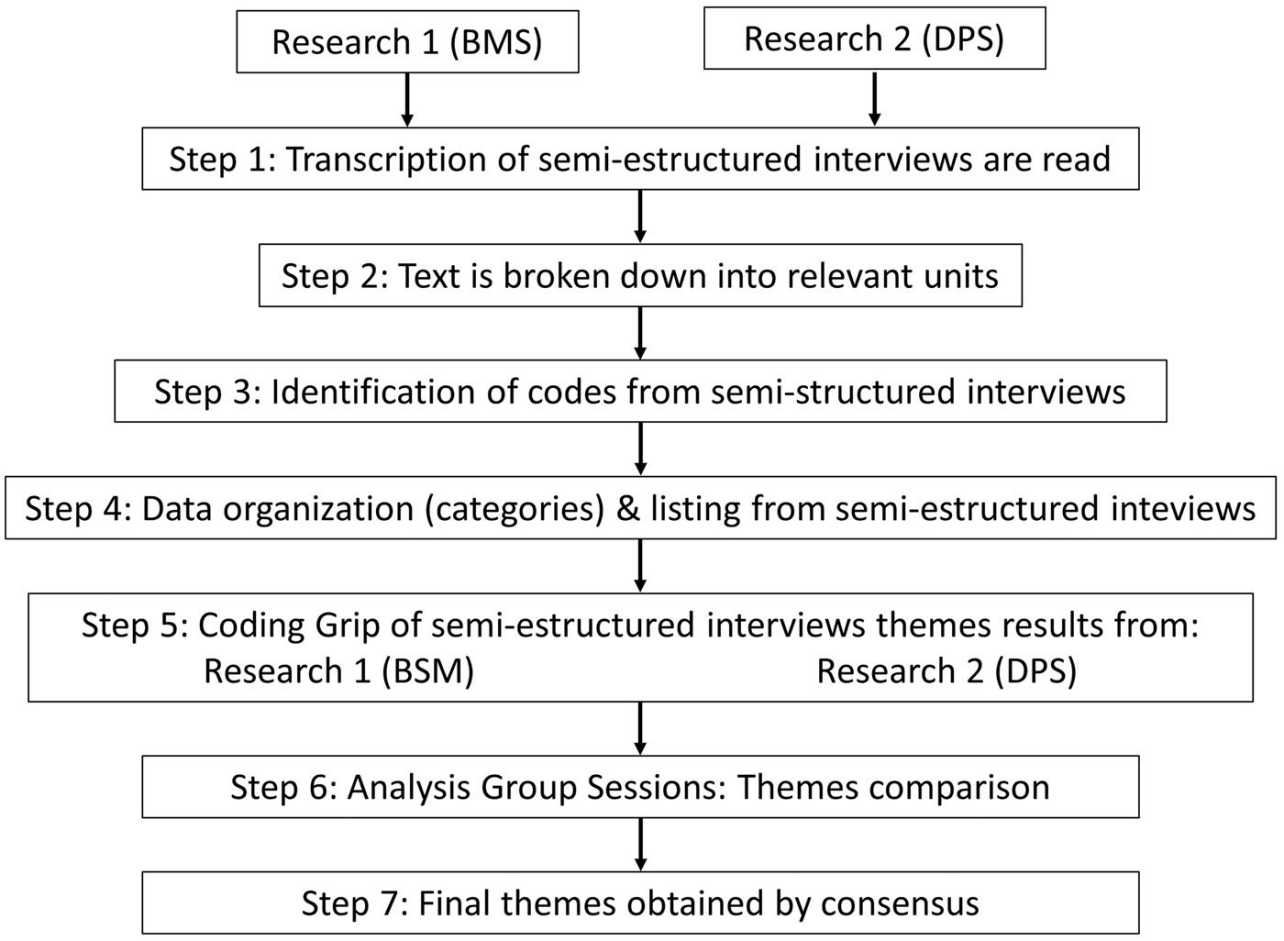
Description of the data analysis process.

The interviews were analysed by means of an inductive thematic analysis (the analysis did not use any predetermined category guide or prior theoretical model) for the identification of the relevant themes obtained from the interviews [28,38]. Full transcripts were made of each in-depth interview and the researchers’ field notes [28,38]. Thematic analysis consisted of identifying text fragments with relevant information to answer the research question. From these narratives, the most descriptive contents (codes) were identified. Subsequently, these units were grouped by their common meaning (categories) and/or similar content [28,38]. At this point, the identification of the themes began, through three phases [39]. In the first phase, "searching for themes", the first proposal of sub-themes and themes identified inductively from the previous codes and categories, reflecting "raw data", was provided. These first themes were temporary. Subsequently, in the "Reviewing Themes" phase, the refinement of the identified themes took place, reviewing the coherence between the theme and the participants' narratives that support it. If the themes did not have sufficient data to justify and support them, they were dropped. Finally, in the "defining and naming themes" phase, the researchers determined the definitive themes, their definition and the information that enabled their identification [39]. Thematic analysis was applied separately to interviews and field notes by BMS and DPC. Joint team meetings were held to combine the results of the analysis and discuss data collection and analysis procedures. In these team meetings, the final themes were displayed, combined, integrated and identified. In case of divergence of opinions, the identification of the theme was based on consensus among the members of the research team (Figure 1). No qualitative software was used for the data analysis.

### Rigour

The techniques performed and application procedures used to control trustworthiness are described in (Table 2) [40].

**Table 2.** Trustworthiness criteria.

Criteria	Techniques Performed and Application Procedures
Credibility	Investigator triangulation: each interview was analysed by two researchers. Thereafter, team meetings were performed in which the analyses were compared, and themes were identified.
	Triangulation of data collection methods: unstructured, semi-structured interviews were conducted, and researcher field notes were kept.
	Member checking: this consisted of asking the participants to confirm the data obtained during the data collection. All participants were offered the opportunity to review the audio and/or video records to confirm their experience. None of the participants made additional comments.
Transferability	In-depth descriptions of the study were performed, providing details of the characteristics of researchers, participants, contexts, sampling strategies, and the data collection and analysis procedures.
Dependability	Audit by an external researcher: an external researcher assessed the study research protocol, focussing on aspects concerning the methods applied and study design.
Confirmability	Investigator triangulation, data collection and analysis triangulation. Researcher reflexivity was encouraged *via* the completion of reflexive reports and by describing the rationale for the study.

## Results

Thirty-one individuals with stroke (eleven female participants, 35.48%) were recruited. The mean age of participants was 64 years (standard deviation, SD: 15), and the mean time since stroke was 38 months (SD: 35). Also, regarding stroke type, seven participants (22.58%) had haemorrhagic stroke, and 24 participants (77.42%) had ischaemic stroke. Moreover, 20 individuals with stroke (64.52%), and 11 individuals (35.48%) presented a moderate and severe score, respectively, on the NIHSS scale. Finally, regarding functional status (using the Barthel index), 11 participants (35.48%) had a mild score, 12 participants (38.71%) had a moderate score and 8 participants (25.81%) obtained a severe score.

Three specific themes emerged from the data analysed: (a) The person behind the patient label, with three categories; (b) The person at the centre of care, with two categories and (c) PCC training, with three categories (Table 3). In the description of each theme, some of the participants' narratives have been reproduced, taken directly from the interviews in relation to the emerging themes.

**Table 3.** Themes and categories that emerged from the participants’ narratives.

Themes	Categories
The person behind the patient label	Recognizing identity
	Sharing your life story
	Holistic care
The person at the centre of care	Participation in decision making
	Building bonds
Training for Person- Centred Care	Emotional and personal competencies
	Professional role
	The healthcare organization as a barrier

### The person behind the “patient” label

#### Recognizing identity

Our participants narrated how professionals do not perceive them as a person, but as a number. They felt that the qualities or aspects that define one’s identity as an individual were undervalued: ‘They see you as a sick person and that's it. Not as a person, who has qualities and a full life before the disease’. (P8)

One of the aspects emphasized was that it is essential to know what is important to the sick person, what defines them, to recognize their way of life and to learn from this, to decide which treatment is the most appropriate: ‘I learn from the doctor, and he learns from me and from my way of living’. (P2)

Among the facilitators described for recognizing identity, our participants highlighted that calling the person by name is proof of the professional's interest. To recognize the other as a person, not as a case number; ‘When I came in, he said, ‘How did you sleep last night, Carmen? I felt recognised, close, and it was a huge boost to my morale to be greeted by my name’. (P17) Knowing everything about the disease, but not knowing (or forgetting) the person’s name is a barrier: ‘Show a minimal interest in you, not just as a patient. Know my name, for me it's the number one priority. They know the map of the veins in my head but if you don't know my name, what good is it? (P2). Another barrier identified was referring to the sick person by bed or file number: ‘I'm not a bed, I'm X[name].’ (P3) ‘In the end they turn people into a number. The sick person is someone occupying a bed, that's what counts’. (P26)

Ultimately, the consequence of losing their identity is that personal treatment is lost, without taking into account the human component, and turning the sick person into a mere cog in a complex machine: ′At the hospital, in the end you feel like a piece of meat, you're a cog in a machine, where they have to shower you, they have to feed you, they have to do something, and they just do it. But nobody ever stops to really ask you about things. I'm not a bundle that gets moved and shaken around.’ (P29)

#### Sharing your life story

Knowing the life story of the sick individual implies knowing their life experiences, their family, their friends, their habits and their environment. In short, knowing what is significant for the person to optimize their recovery: ′They need to ask about this, because they need to know about my life, as they are going to treat me based on that. ’ (P19)

Within their life story, participants considered that suffering a stroke was the hardest episode of their lives. They defined it as a traumatic situation, leaving a profound mark, which was devastating and very tough: ′Devastating I would say, to put it in a word. It took away my whole life.’ (P9) Stroke changes your whole life, breaks down everything, changes all your habits, rhythms of life, uprooting and removing everything that is important in people's lives: ′The stroke itself was a tsunami. Because it's not just the neurological and physical effects, it breaks down your whole life, it tears everything away from you. ’ (P10)

Our participants narrated how the person who has suffered a stroke begins a new chapter in their story. The person who once was ′dies’ and a new version is created that needs to learn another life: ′The person you were dies and you become a different person. You go through a mourning, your usual self has just died, and you are building a new one, but first you must bury the self who has died. ’ (P9)

While building their new life, participants describe the great emotional impact of stroke, the need to feel the support and concern of professionals, as they are lost, helpless, defenceless, vulnerable and alone: ′If professionals do not consider the emotional component of that person, they are undermining the recovery process. It is fundamental, critical, and mandatory. ’ (P1), ′When you are very sick, you are sensitive and vulnerable, you feel very alone. It's like being a child. ’ (P19)

In their life stories, the participants narrated how the hospital environment marked them. The hospital was perceived as a harsh experience, which had a great emotional impact on them as they had to live with other sick people, witness the deterioration of other people and experience the emotional moments of other families: ′It was a tough environment, I was one of the lucky ones because there were people who were much worse off, you saw it every day: being in an environment where you see sick people all around you is not good for your recovery. ’(P7)

#### Holistic care

Our participants narrated how the disease affected all dimensions of the person. Integrating all these dimensions and having a global and holistic perspective of the person influences the individual’s rehabilitation: ′To integrate the physiotherapy session into the patient's life. Considering the overall picture, it is not a person with stroke, but a person with more affected areas. The patient is considered like islands (…) as if the human being is made up of different pieces, when the human being is actually a whole (…). ’ (P1)

These dimensions to be considered include individual tastes and preferences (such as food), care of body image, integrating their customs and habits in the recovery process: ′This week I don't want to eat peas, is that so serious? I can understand that there are limited resources, and they're not going to make you a dinner you're craving. But they might listen to you. ’(P29), ′I don't want my hair to look like a crazy woman. I just ask you to brush it, do you think I want to ask you? I want to do it myself, but I can’t. ’ (P3)

These dimensions clash with the centre's healthcare organization, making their requests difficult or cumbersome. In contrast, the presence and accompaniment of professionals during their recovery was positively valued.: ′They don't always have to do something, sometimes it's just being there, that' s all. It's important for them to be around.’ (P2). For our participants, when the professionals showed an interest in knowing part of their lives, their family and work relationships, their home and how the disease may have affected them, this improved the therapeutic relationship and the establishment of a bond.

The protection of privacy and the recognition of spiritual beliefs were described as two key elements of their relationship. Privacy goes beyond the symptoms of the disease and requires a great deal of tact on behalf of the professional. Moreover, recognizing the spirituality of the person generates a sense of security and encouragement for the participant. ′Religion, spirituality, beliefs, whatever, Buddhist, Muslim, Catholic… helps a lot. It gives security. And recognising and respecting that helps the patient a lot. Very much. ’ (P15)

### The person at the centre of care

#### Participation in decision making

Participation is a key element in making the person feel present and integrated in their process of rehabilitation and recovery. The person must participate in the decisions that affect their process, with their opinions being taken into consideration:

*When the professional who approaches you has that in mind, it makes you feel that your opinion matters and that you have the ability to make decisions and think. Some professionals have made me understand that I had a lot to contribute to my own process, whereas others haven't conveyed this to me*. (P9)

A participatory scenario is proposed in which both the participant and the professionals view the process and make decisions together. This participatory space is a space for dialogue and joint thinking, by including the sick person: ′They don't just come to lecture you or tell you what's good for you, they involve you in things.’ (P9). These spaces should also be a place where doubts can be resolved and where trust can be fostered between the professional and the individual: ′Having doubts, and being able to share them, is a sign of good professional treatment’ (P4).

The participants described how sharing the treatment plan with them, informing them, incorporating their opinion, establishing their own limits is a sign of respect and justice for the person. Decisions are consensual, considering the person's personal goals, and avoiding imposing what they believe is good for the person with stroke from the professionals' perspective: ′Not to be imposed on or told what's good for me from the outside. At some point they should ask me, shouldn't they?’ (P9)

Among the obstacles to the participation of people with stroke, the following were described: (a) not informing the participant of changes in treatment, decisions on the rehabilitation plan and results of tests performed; and (b) keeping the participant out of conversations held among different specialists or groups of professionals: ′It's no good for them to come to see you, evaluate you and then go to a corner and talk among themselves. Explain to me what's going on because I want to know.’ (P29)

This caused the participants to feel a loss of control or to feel that their life was in someone else's hands. They felt that they were passive subjects, that they had no control or power to decide about their own recovery which is being experimented with by others: ′I remember feeling like I was in someone else's hands. I was not at all comfortable with the feeling that my fate was in other people's hands and not my own." (P9) "As much as I trusted them, it felt like I was a guinea pig.’ (P10)

#### Building bonds

Knowing different aspects of the professionals' lives facilitates the building of interpersonal relationships and improves the bond between people with stroke and the professionals. Our participants reported how being able to get to know personal aspects (habits, family, etc.) of the professionals made them feel that they were on the same level and there was no hierarchy between them: ′Often they would tell me their things, their problems, and I felt that they put me on their level. I felt that I was closer to them’ (P14) Our participants described how a treatment similar to a friendly relationship generates a greater bond: ′A friendship is created, so to speak. You create a bond between the two of you (…) You become attached to a person, and there is always a greater consideration when there is a relationship’ (P18).

The main barrier to building links is the positioning of professionals at a higher (hierarchical) level that is inaccessible to the sick person: ′It's worse the higher they are, they are more uptight (…) Some rehabilitation doctors, they are like on another level. Others can't reach that level. But neither can the patients’ (P3). This positioning is perceived by the participants as a safeguard of their authority as professionals:

*Keeping a certain distance safeguards authority. Those who must command, based on competent authority, avoid excessive proximity to those who receive it. If the physician thinks that maintaining this authority is more important than maintaining credibility with the patient, he is radically mistaken. In the case of my doctor, he was able to overcome this risk very well and I am extremely grateful to him for that.* (P10)

Another barrier identified was professionals who believed that they knew more about the person, their experiences and their expectations of recovery, based on their profession and technical knowledge. This generates an invisible barrier affecting their treatment: *Nobody there admits they are wrong*. They don't, they are doctors, and you know nothing about what is happening to yoú (P3). ′The roles are very different. There is an invisible barrier that you notice right away; those who are in charge and those who are not in charge, those who know and those who don't know…’ (P10).

### Training for person-centred care

#### Emotional and personal competencies

The participants recounted how professionals need to know and have the skills to manage their own emotions. This is because their emotions influence the care of sick people. Professionals are faced with difficult situations and great pressure that can overwhelm them personally. It is therefore necessary for professionals to acquire the necessary tools for this management: ′The professional can be outstanding, but we have to take into account whether the emotional aspect is balanced’ (P1). ′Sometimes they are overwhelmed with work and their patience runs out. That does not mean that they are a bad professional. ’ (P27)

Moreover, the situation of the individuals with stroke can affect health professionals emotionally because they suffer due to not being able to fully rehabilitate or recover the person. Our participants recounted how this suffering leads to professionals protecting themselves by creating a sort of armour to contain their emotions:

They are not made of stone; they have to put on an armour to protect themselves from us and from what happens to us. They must protect themselves and take care of themselves, they can't worry you so much. I think they need a shell because if not every day like this, year after year, it would be very hard (P3).

Our participants pointed out that rehabilitation professionals must have certain personal skills to care for the person with stroke. Among others; understanding, benevolence and sensitivity. These competencies or skills are the ones that ensure that healthcare work has a high human quality:

*I noticed a lot of kindness in the healthcare professionals. During that process, during that time that I was so close to them, I discovered that these people really have a special human quality, from the most qualified to the least qualified, to the most skilled, to the most experienced.* (P9)

#### The role of the professional

From the participants’ perspective, professionals and their interventions play a fundamental role in PCC. Concretely, by protecting the person’s dignity, the recognition of their individuality and helping them to regain their confidence: ′You have to regain your life and your dignity, at that moment you don't have it or it is very diminished. They help you to recover it step by step’ (P10). Another aspect is concern for the participant, putting their care and recovery before other factors such as healthcare organization, administrative procedures, etc.:

*The difference between a farmer and a doctor (…). You can find doctors who are only interested in filling out forms and then they listen to the patient. The farmer, first, worries about the cattle, doesn't he? It does not occur to a stockbreeder to say that today is a holiday, and that the cattle should be left to fend for themselves. No, his first concern is his cattle. To take care of them. Essentially that's what it is, they should worry more about the sick. That's the main priority*. (P13)

Another aspect that was highly valued by participants was feeling seen and recognized by the professional. Thus, they felt considered when they were recognized by professionals, and when importance was given to their perspective and opinion: ′You feel that they are recognising you and it makes you happy and think that you are not so alone. What they [professionals] show is that they care and they're going to use all their knowledge and experience to help you. And that makes us all feel good’ (P2). In addition, participants valued the professionals' ability to provide reassurance, support, and companionship: ′Having them reassure you, it's almost more important that they do all the tests for you. To be told 'Calm down, we’re by your side, don't worry, you’re not going to be aloné (P23).

Empathy is also valued as an element in PCC. It means that the professional understands the person, putting oneself in the individual's shoes and trying to learn from the participant's perspective: ′Empathizing to know what they think from another person's point of view is the only way to be able to learn. When you empathise, you learn how others think’ (P6). In addition, investing time in conversations and continuous dialogue is essential to be able to understand and empathize with sick people. For the participants, moments of dialogue are times when they show that they have an opinion, criteria, that they can talk and think about other topics. It helps them to get out of their role of feeling labelled as a patient:

′*There were times when the doctor would come on rounds, and he would just come to chat. I am very grateful to him for that. I am a patient, but I have criteria, he can give his opinion on everything. One of the funniest things was to talk about things that happened at the hospital*. (P29)

#### The healthcare organization as a barrier

Participants described the automation of work, the automatic application of protocols and the limited time spent in contact with individuals with stroke as barriers to training professionals for PCC. Automation is described by the participants as the systematic performance of tasks, with limited dedication to the treatment and care of the person and towards the care process: ′It's not something that is inherent to the professional. It was weird, because from one day to the next, they would turn into robots, and they would turn you, into meat or the number of diapers you have to change that night’ (P2). Our participants described this as feeling like being on an assembly line: ′They have to do a job and they have a set time to carry it out. So, that personal relationship that might be interesting is discarded. It's hard, because in the end you become a piece of meat that's on an assembly liné (P29). The automatic use of protocols or work procedures may not be suited to the real needs of people with stroke. For our participants, care was depersonalized by applying the procedure without including or considering the person’s specific situation: ′They don't pay any attention to the patient, they just follow the protocol. I had the feeling that they asked me and ordered things because it was in the manual, not because they were interested. It was like a script’ (P20). The participants failed to understand the professionals' lack of time, understanding that they are the focus and the reason for the professionals' work, their occupation: ′They don't have time for me, busy all day…I don't know what for, I'm the one who' s sick’ (P6). In addition, perceiving that professionals were always "rushing around" and asking them questions in a hurry, and not having time to dedicate to them, conveys a feeling of being overwhelmed to the person receiving care: ′The truth is that some people, they just rush around and that's it. They don't even look at yoú (P12).

## Discussion

Our results show the importance of applying a PCC model of care for people with stroke. Aspects such as recognizing the identity of the person and their dimensions, their life story, encouraging their participation in decision making, building links between professionals and people undergoing rehabilitation, the professional role, together with the barriers that influence care, should be taken into account in the application of PCC.

In terms of knowing the person underlying the ′patient’ label, previous studies have described that understanding and synchronizing the personal dimensions of the person with stroke help to involve the person in the care process and facilitates adaptation [18]. Promoting the caregiver's own identity [41,42] requires encouragement and empowerment of individuals with stroke to regain a sense of control and self-efficacy [43]. Tonelli and Sullivan [43] state that the healthcare professional should know the people with disease to be able to answer the question: ‘What would you do if you were me?’ These authors start from a model that recognizes professionals as individuals and requires them to understand persons with stroke as individuals, through a direct and particular knowledge of each person cared for [43].

Our results illustrate the relevance of knowing the participant's life story. This means contributing to the quality of professional intervention through respectful and responsive care to the individual's needs, ensuring that the individual values of the person being cared for are respected. Previous studies describe how clinical care should strengthen the capacity to receive the stories that the persons cared for provide about themselves, through active listening to their life story [44]. Recognizing and interpreting individuals' life stories allows the clinical gaze to be extended to personal and vital elements beyond the role of patient [7,10,45].

Our results show the need to integrate a comprehensive vision of people with stroke into clinical practice. This holistic integration of care in individuals with stroke has been described by previous studies that analysed the suitability of applying PCC in the rehabilitation process from a vision of global understanding of the person [3,6,11]. Previous studies highlight the importance of showing interest in the person as a whole, gaining an understanding of their psychological and emotional health, spiritual and existential problems, living conditions, financial situation, social support system and culture [19], providing a space in which to express their values, fears and aspirations as an opportunity to improve perceived satisfaction [15]. Personal dimensions, such as daily routines, social obligations and spirituality, should therefore be included [18]. Spirituality in individuals with stroke should also be considered within the person's globality, as it has direct repercussions on the perception of quality of life of individuals with stroke treated in the health service [46,47].

In rehabilitative health care, an individualized approach recognizes the uniqueness of the person, allowing the world to be interpreted from the person's perspective [5]. Previous studies have described the need for ongoing reflection on health care that integrates what is meaningful to the person during individualized care [48,49]. It is necessary to know the importance that each individual with stroke gives to their own disease-recovery [3], and to their character or personality in order to cope with treatment [3,14,20]. Previous studies describe how the knowledge acquired regarding the individual experiences of each individual after suffering a stroke should be the key to a PCC based on each person's reality [50], finding that the person is facing a new and difficult reality of biographical interruption of great impact that generates the need for relational orientation towards care [3].

Another key point is to understand, from the perspective of people with stroke, those professional characteristics that facilitate the application of PCC. Talen *et al.* [45] and Colombo [51] described that certain behaviours and attitudes of the health professional, such as providing more information to the individuals with stroke, paying greater attention to the person and their social context and paying attention to feelings and emotions, facilitated the connection between professionals and individuals and improved PCC. Previous studies coincide with our results, where showing concern for the individuals, interest in their experience and showing empathy improve the satisfaction of those cared for [3,7,48,52]. In addition, it is necessary to encourage continuous communication including everyday issues for the person, generating tranquillity and reinforcing the individual’s dignity [50,53,54].

In the PCC model, the person plays an active role, and should be considered as a partner with capabilities and resources to contribute to their care [55]. Previous studies have shown that during rehabilitation, it is necessary to identify life goals and interventions [4], understanding which ones are meaningful to the individual [48]. This would enable the establishment of person-centred goals in a collaborative and egalitarian manner, moving away from pre-established roles [13] and respecting the individual's limits [56]. The fact that the person is the protagonist of their intervention generates empowerment and improves the meaning of life when they feel supported in the goals that they themselves have defined [7,49].

Facilitating goal setting according to the PCC model for the individual who has suffered a stroke has a positive influence, maintaining hope and a sense of momentum in recovery [3,20]. Respect for the individual as a person goes beyond respect for the person’s choice, and it is paramount for ensuring the individual's autonomy. Partnership with healthcare professionals together with transparency are requirements for individuals with stroke to be able to accept and participate in their own process [3,15,43,57]. This associative or horizontal relationship is based on a therapeutic alliance in which individuals feel trust [58], facilitating information, knowledge, dialogue and decision making in a cooperative and reciprocal manner between individuals with stroke and professionals [56]. In this manner, a space for PCC practice can be created, offering options and opportunities for individual involvement through participatory decisions from relationships of professional–person equality [14].

Even though PCC has become widespread, it is necessary to deepen our understanding of the measures required for its complete integration into clinical healthcare practice [21]. One pillar of this integration is the training of healthcare professionals to perform PCC in people with stroke. Previous studies describe how education and training increase the commitment of professionals, and the recognition of the person's autonomy [45,48,56,58]. For Terry and Kayes [3], the personalized and committed PCC of stroke patients was described in relational terms with professionals, even beyond their skills or knowledge. In addition, it is therefore necessary for professional training to include emotional management and distress management, through the acquisition of professional skills. This makes it easier for professionals to provide greater support and assistance to individuals and to provide closer and more humane care to people with stroke [44].

Even though health systems should ensure quality of care based on their ability to meet users' expectations [15,59], there are difficulties in implementing PCC [3,8,60]. Factors such as workload, pressure or time constraints become barriers in daily practice [3,8]. American Geriatrics Society Expert Panel on Person-Centred Care [8] points out that one of the main obstacles to the implementation of PCC is that most health care systems are still based on traditional approaches to clinical practice. This means that in clinical situations with high-care pressure, or under time constraints, PCC cannot be applied in Depth [60]. Moreover, its implementation requires an extra workload for healthcare professionals, and it does not promote the investment of resources in PCC in the long term, despite the increased satisfaction of patients and families with PCC and its benefits in clinical practice [3,60].

In the future, it would be necessary to continue implementing and studying PCC, to obtain the commitment and interest of health care professionals to integrate it into their clinical practice, and to obtain the investment of resources from health care managers and administrations to provide health care professionals with the means to do so. Table 4 shows the recommendations for health professionals/policy makers/researchers.

**Table 4.** Recommendations for health professionals/policy makers/researchers.

Researchers	Health professionals	Policy makers
Reflection and dissemination of the definition and progress of the PCC^a^ model.	Commitment and interest in the PCC^a^ model	To introduce specific curricular competencies in PPC^a^ within the academic training itinerary of health professionals.
Facilitate the understanding of barriers and facilitators in the development of PCC^a^ in various healthcare settings.	Adaptation to the change of roles acquired in other health care models, enhancing doctor–patient collaboration.	Generate and promote health policies that place the person at the centre of clinical care.
Disseminate PCC^a^ experiences after the implementation of the PCC^a^ model.	Integration of PCC^a^ into daily clinical practice and evaluation of the implementation of the therapeutic plan in real healthcare contexts.	Provide sufficient economic, professional and logistical resources for the optimal development of PCC^a^.
To introduce participants (individuals with stroke and their families) as active agents in the research project design process.	Learning and training in personal, relational and communication skills and competencies specific to the PCC^a^.	Coordination of the implementation of PCCa in the long term, and investment in resources, avoiding the punctual, anecdotal implementation of PCC^a^.
To generate common spaces between researchers, managers, health professionals, institutions and administrations, together with individuals with stroke and their families, in order to know the needs, expectations and potentialities of the implementation and development of PCC^a^		

^a^Person-centred care.

## Conclusions

People who have suffered a stroke positively value health care is based on the PCC model, in which their individual circumstances and characteristics are respected in clinical care.

Our results highlight the importance of recognizing the person’s identity, seeing them beyond their illness and valuing their wishes and expectations. It is essential to recognize the influence of their life history as well as the need to rebuild themselves after the stroke. It is necessary to adopt a comprehensive vision of individuals with stroke through holistic care that includes their tastes, preferences, customs, habits and beliefs in their care process. The person must be at the centre of their intervention, respecting both their preferences in their intervention plan and their limits. For this, it is still necessary to train health professionals and managers in emotional and personal skills that facilitate attitudes and skills such as listening or empathy. Also, organization aspects must be improved, such as the protocolization of interventions or automation of work, to continue promoting and developing the CPP model in individuals with stroke during their rehabilitation.

## Limitations

This study has limitations concerning generalizability. Also, stroke evolution and types may underlie differences in disease experience and how it affects daily life, perspectives of decision-making and conflicts regarding autonomy. Moreover, in this study, individuals with mild cognitive impairment and aphasia were excluded. This could limit access to the perspective of vulnerable individuals who would genuinely benefit from PCC. Furthermore, this study did not investigate the experience of individuals with stroke on the basis of ethnicity or socioeconomic background. Studies focussing on these groups of people would need to be developed. Moreover, regarding sample size sufficiency, previous studies [34,35] reported that the sample size justification in qualitative health research is limited and defining sample size a priori is inherently problematic in the case of inductive, exploratory research. Also, Sebele-Mpofu [36] describe how the definition of the concept of saturation can vary (theoretical saturation, thematic saturation, data saturation, meaning saturation), depending on the qualitative design chosen, sampling strategy (purposeful, convenience, theoretical) and the data collection instrument used. Due to this great variability of criteria, the authors chose a proposal to establish the sample size based on empirical criteria, where a study of the number of interviews necessary to obtain the maximum percentage of the participants' narrative content would have been carried out. Therefore, the method chosen to determine the sample in our study was the proposal by Turner-Bowker *et al.* [37] which involves providing a starting point for the a priori estimation of sample size for a specific type of qualitative research with qualitative concept elicitation, and also, using an evidence-based approach to generate these recommendations. Finally, it is important to consider the perspectives of the individuals’ partners for a more comprehensive analysis of PCC during the rehabilitation process after stroke.

## Supplementary Material

Supplemental Material

## Data Availability

The data that support the findings of the study are available on request from the corresponding author, upon reasonable request. The data are not public due to ethics restrictions.
