# Supplementary material for: Person-centred care in individuals with stroke: a qualitative study using in-depth interviews
Source: Ann Med. 2022 Aug 5;54(1):2167–80. doi: 10.1080/07853890.2022.2105393 (PMC9359161; doi:10.1080/07853890.2022.2105393)
Supplement: Supplemental Material [file IANN_A_2105393_SM3270.docx]

**Supplemental Table 1.** COREQ check list

| No | Item | Guide questions/description | Observations |
| --- | --- | --- | --- |
| **Domain 1: Research team and reflexivity** |  |  |  |
| Personal Characteristics |  |  |  |
| 1. | Interviewer/facilitator | Which author/s conducted the interview or focus group? | Page 10 |
| 2. | Credentials | What were the researcher's credentials? *E.g. PhD, MD* | Page 8 |
| 3. | Occupation | What was their occupation at the time of the study? | Page 8 |
| 4. | Gender | Was the researcher male or female? | Page 8 |
| 5. | Experience and training | What experience or training did the researcher have? | Page 8 |
| Relationship with participants |  |  | Page 8 |
| 6. | Relationship established | Was a relationship established prior to study commencement? | Page 8 |
| 7. | Participant knowledge of the interviewer | What did the participants know about the researcher? e*.g. personal goals, reasons for doing the research* | Page 8 |
| 8. | Interviewer characteristics | What characteristics were reported about the interviewer/facilitator? e.g. *Bias, assumptions, reasons and interests in the research topic* | Page 8 |
| **Domain 2: study design** |  |  |  |
| Theoretical framework |  |  |  |
| 9. | Methodological orientation and Theory | What methodological orientation was stated to underpin the study? *e.g. grounded theory, discourse analysis, ethnography, phenomenology, content analysis* | Page 7 |
| Participant selection |  |  |  |
| 10. | Sampling | How were participants selected? *e.g. purposive, convenience, consecutive, snowball* | Page 8 |
| 11. | Method of approach | How were participants approached? e*.g. face-to-face, telephone, mail, email* | Page 9, 10 |
| 12. | Sample size | How many participants were in the study? | Page 9, 12 |
| 13. | Non-participation | How many people refused to participate or dropped out? Reasons? | Page 9 |
| Setting |  |  |  |
| 14. | Setting of data collection | Where was the data collected? e*.g. home, clinic, workplace* | Page 9 |
| 15. | Presence of non-participants | Was anyone else present besides the participants and researchers? | Page 9 |
| 16. | Description of sample | What are the important characteristics of the sample? *e.g. demographic data, date* | Page 11,12 |
| Data collection |  |  |  |
| 17. | Interview guide | Were questions, prompts, guides provided by the authors? Was it pilot tested? | Page 9, 10, table 1 |
| 18. | Repeat interviews | Were repeat interviews carried out? If yes, how many? | Page 9, 10 |
| 19. | Audio/visual recording | Did the research use audio or visual recording to collect the data? | Page 9, 10 |
| 20. | Field notes | Were field notes made during and/or after the interview or focus group? | Page 9, 10 |
| 21. | Duration | What was the duration of the interviews or focus group? | Page 9, 10 |
| 22. | Data saturation | Was data saturation discussed? | Page 9, 27 |
| 23. | Transcripts returned | Were transcripts returned to participants for comment and/or correction? | Page 11, table 2 |
| **Domain 3: analysis and findings**z |  |  |  |
| Data analysis |  |  |  |
| 24. | Number of data coders | How many data coders coded the data? | Page 11 |
| 25. | Description of the coding tree | Did authors provide a description of the coding tree? | Page 10, 11, figure 1, table 3 |
| 26. | Derivation of themes | Were themes identified in advance or derived from the data? | Page 10, 11, figure 1, table 3 |
| 27. | Software | What software, if applicable, was used to manage the data? | Page 11 |
| 28. | Participant checking | Did participants provide feedback on the findings? | Page 11, table 2 |
| Reporting |  |  |  |
| 29. | Quotations presented | Were participant quotations presented to illustrate the themes / findings? Was each quotation identified? e*.g. participant number* | From page 11 to page 22 |
| 30. | Data and findings consistent | Was there consistency between the data presented and the findings? | Page 11-22 |
| 31. | Clarity of major themes | Were major themes clearly presented in the findings? | Page 11-22, table 3 |
| 32. | Clarity of minor themes | Is there a description of diverse cases or discussion of minor themes? | Page 11-22, table 3 |

**Supplemental Table 2.** SRQR check list

| No | Item | Guide questions/description | Observations |
| --- | --- | --- | --- |
|  | **Title and abstract** |  |  |
| S1 | Title | Concise description of the nature and topic of the study Identifying the study as qualitative or indicating the approach (e.g., ethnography, grounded theory) or data collection methods (e.g., interview, focus group) is recommended | Page 3 |
| S2 | Abstract | Summary of key elements of the study using the abstract format of the intended publication; typically includes background, purpose, methods, results, and conclusions | Page 3,4 |
|  | **Introduction** |  |  |
| S3 | Problem formulation | Description and significance of the problem/phenomenon studied; review of relevant theory and empirical work; problem statement | Page 5-7 |
| S4 | Purpose or research question | Purpose of the study and specific objectives or questions | Page 7 |
|  | **Methods** |  |  |
| S5 | Qualitative approach and research paradigm | Qualitative approach (e.g., ethnography, grounded theory, case study, phenomenology, narrative research) and guiding theory if appropriate; identifying the research paradigm (e.g., postpositivist, constructivist/interpretivist) is also recommended; rationale | Page 7 |
| S6 | Researcher characteristics and reflexivity | Researchers’ characteristics that may influence the research, including personal attributes, qualifications/experience, relationship with participants, assumptions, and/or presuppositions; potential or actual interaction between researchers’ characteristics and the research questions, approach, methods, results, and/or transferability | Page 7, 8 |
| S7 | Context | Setting/site and salient contextual factors; rationale | Page 8, 9 |
| S8 | Sampling strategy | How and why research participants, documents, or events were selected; criteria for deciding when no further sampling was necessary (e.g., sampling saturation); rationale | Page 8 |
| S9 | Ethical issues pertaining to human subjects | Documentation of approval by an appropriate ethics review board and participant consent, or explanation for lack thereof; other confidentiality and data security issues | Page 7 |
| S10 | Data collection methods | Types of data collected; details of data collection procedures including (as appropriate) start and stop dates of data collection and analysis, iterative process, triangulation of sources/methods, and modification of procedures in response to evolving study findings; rationale | Page 9, 19, table 1 |
| S11 | Data collection instruments and technologies | Description of instruments (e.g., interview guides, questionnaires) and devices (e.g., audio recorders) used for data collection; if/how the instrument(s) changed over the course of the study | Page 9, 19, table 1 |
| S12 | Units of study | Number and relevant characteristics of participants, documents, or events included in the study; level of participation (could be reported in results) | Page 11, 12 |
| S13 | Data processing | Methods for processing data prior to and during analysis, including transcription, data entry, data management and security, verification of data integrity, data coding, and anonymization/deidentification of excerpts | Page 10, 11, figure 1 |
| S14 | Data analysis | Process by which inferences, themes, etc., were identified and developed, including the researchers involved in data analysis; usually references a specific paradigm or approach; rationale | Page 10, 11, figure 1 |
| S15 | Techniques to enhance trustworthiness | Techniques to enhance trustworthiness and credibility of data analysis (e.g., member checking, audit trail, triangulation); rationale | Page 11, table 2 |
|  | **Results/Findings** |  |  |
| S16 | Synthesis and interpretation | Main findings (e.g., interpretations, inferences, and themes); might include development of a theory or model, or integration with prior research or theory | Page 11-22, table 3. |
| S17 | Links to empirical data | Evidence (e.g., quotes, field notes, text excerpts, photographs) to substantiate analytic findings | Page 11-22, table 3. |
|  | **Discusssion** |  |  |
| S18 | Integration with prior work, implications,  transferability, and contribution(s) to the field | Short summary of main findings; explanation of how findings and conclusions connect to, support, elaborate on, or challenge conclusions of earlier scholarship; discussion of scope of application/ generalizability; identification of unique contribution(s) to scholarship in a discipline or field | Page 22-26, table 4 |
| S19 | Limitations | Trustworthiness and limitations of findings | Page 27, 28 |
|  | **Other** |  |  |
| S20 | Conflicts of interest | Potential sources of influence or perceived influence on study conduct and conclusions; how these were managed | Page 28, 29 |
| S21 | Funding | Sources of funding and other support; role of funders in data collection, interpretation, and reporting | Page 28, 29 |
